# Changes in Life Satisfaction and Self-Esteem During Neoadjuvant Chemotherapy in Women with Breast Cancer: A Prospective Observational Study

**DOI:** 10.3390/cancers18091421

**Published:** 2026-04-29

**Authors:** Magdalena Konieczny, Dorota Kiedik, Jolanta Sawicka, Izabela Gąska, Dorota Bądziul, Elżbieta Kaczmar, Agnieszka Kiedik, Łukasz Rypicz

**Affiliations:** 1 Medical Institute, Jan Grodek State University in Sanok, 38-500 Sanok, Poland; magdalenakonieczny@wp.pl (M.K.); jsawicka@up-sanok.edu.pl (J.S.); igaska@up-sanok.edu.pl (I.G.); ekaczmar@up-sanok.edu.pl (E.K.); 2 Department of Public Health, Division of Public Health, Faculty of Health Sciences, Wroclaw Medical University, 50-367 Wroclaw, Poland; dorota.kiedik@umw.edu.pl; 3 Department of Medical Biology, Institute of Medical Sciences, Medical College of Rzeszow University, Rejtana 16 C, 35-959 Rzeszów, Poland; dbadziul@ur.edu.pl; 4 Faculty of Medicine, Medical University of Gdansk, 80-210 Gdansk, Poland; agnieszka.kiedik@gumed.edu.pl

**Keywords:** breast cancer, neoadjuvant chemotherapy, life satisfaction, self-esteem

## Abstract

**Background/Objectives:** Neoadjuvant chemotherapy is widely used in the treatment of breast cancer and improves oncological outcomes; however, its impact on patients’ psychosocial well-being remains insufficiently explored. This study aimed to assess changes in life satisfaction and self-esteem in women with breast cancer undergoing neoadjuvant chemotherapy. **Methods:** A prospective observational study was conducted among 211 women diagnosed with breast cancer and treated with neoadjuvant chemotherapy. Life satisfaction was assessed using the Satisfaction with Life Scale, and self-esteem was measured with the Rosenberg Self-Esteem Scale. Assessments were performed one week before initiation of chemotherapy and three weeks after its completion. Statistical analyses included descriptive statistics, the Wilcoxon signed-rank test, and Spearman correlation analysis. **Results:** A significant decrease in life satisfaction was observed after chemotherapy (mean 21.6 vs. 18.7; *p* < 0.001), indicating a shift from slight satisfaction to slight dissatisfaction. Self-esteem also significantly decreased (29.4 vs. 27.8; *p* < 0.001), although it remained within the average range. A decline in life satisfaction was reported in 60.7% of patients and a decrease in self-esteem in 50.2%. A significant positive correlation between life satisfaction and self-esteem was observed both before and after treatment. Financial status significantly differentiated both outcomes after chemotherapy. **Conclusions:** Neoadjuvant chemotherapy is associated with a significant decline in life satisfaction and self-esteem in women with breast cancer, highlighting its psychosocial impact. These findings support the need for routine assessment of psychological well-being and the integration of psycho-oncological support into comprehensive cancer care. Because the post-treatment assessment was performed shortly after chemotherapy completion, the findings primarily reflect short-term changes during treatment phase.

## Simple Summary

Breast cancer treatment has improved survival, but it can also affect how patients feel about their lives and themselves. Chemotherapy given before surgery is an important part of treatment, yet its impact on emotional well-being is still not fully understood. This study aimed to examine how neoadjuvant chemotherapy influences life satisfaction and self-esteem in women with breast cancer by comparing their condition before and after chemotherapy. The findings showed that many patients experienced a decline in both life satisfaction and self-esteem following treatment. These results highlight the need to pay greater attention to the psychological aspects of cancer care. Understanding these changes may help improve patient support and encourage the integration of psychological care into routine cancer treatment.

## 1. Introduction

Breast cancer is the most frequently diagnosed malignant neoplasm among women worldwide, accounting for approximately 2.3 million new cases and 670,000 deaths annually [1]. This represents a major global health burden, as it remains the most commonly diagnosed cancer among women in 158 out of 185 countries, including Poland [2]. According to the most recent data from the National Cancer Registry (Krajowy Rejestr Nowotworów, KRN), in 2023 there were 21,924 new cases of breast cancer and 6827 deaths among women in Poland. This corresponds to over 23% of all newly diagnosed cancers and approximately 15% of cancer-related deaths in women [3]. Advances in diagnostics and treatment have contributed to a steady improvement in survival outcomes among patients with breast cancer [1]. Breast cancer is a heterogeneous disease influenced by genetic, hormonal, and environmental factors. Its clinical course and treatment response may differ depending on molecular subtype, including hormone receptor-positive, *HER2*-positive, and triple-negative disease. Management is multidisciplinary and may include surgery, systemic therapies, and radiotherapy, while neoadjuvant chemotherapy constitutes an important therapeutic strategy in selected patients. Women diagnosed with breast cancer undergo various therapeutic modalities. Although these treatments reduce mortality, they are associated with side effects that may lead to deterioration in quality of life (QOL) and alterations in self-perception. Chemotherapy, as a key component of breast cancer treatment, is associated with numerous adverse effects, including chronic fatigue, nausea, cognitive impairment, and changes in physical appearance, all of which may significantly affect QOL, life satisfaction, and self-esteem [4,5]. An important component of contemporary breast cancer management is neoadjuvant chemotherapy, administered prior to surgical treatment to reduce tumor size, increase the likelihood of breast-conserving surgery, and enable assessment of tumor sensitivity to systemic therapy [6,7]. Numerous studies have demonstrated that achieving a pathological complete response (pCR) following neoadjuvant chemotherapy is associated with improved prognosis, particularly in biological subtypes such as triple-negative and *HER2*-positive breast cancer [7,8]. At the same time, the neoadjuvant treatment period constitutes a significant psychological burden, as it involves coping both with a cancer diagnosis and with the adverse effects of intensive chemotherapy prior to surgery. Evidence suggests that during this period, patients experience increased psychological distress, reduced QOL, and fluctuations in self-esteem and life satisfaction [9]. Consequently, increasing attention is being paid to the psychosocial aspects of cancer and its treatment, including subjective psychological well-being during systemic therapy.

One of the key cognitive components of well-being is life satisfaction, defined as a global, subjective evaluation of one’s overall QOL in relation to individual standards [10]. Studies conducted in populations of women with breast cancer have demonstrated that life satisfaction is significantly reduced during periods of intensive treatment, and its level is influenced by psychosocial factors such as social support, economic status, and the degree of adaptation to the disease [11]. Another important psychological construct is self-esteem, understood as a relatively stable, global evaluation of one’s own worth [12]. Research findings indicate that self-esteem is an important factor facilitating recovery and return to daily functioning in patients with breast cancer [13]. In the context of breast cancer, self-esteem is particularly vulnerable to decline due to changes in body image, hair loss, functional limitations, and disturbances in sexual functioning, which frequently accompany chemotherapy [5]. Studies published in the international literature indicate that lower self-esteem is associated with poorer psychological adjustment, increased depressive symptoms, and reduced QOL among women treated for breast cancer [14]. At the same time, the protective role of body acceptance and intrapsychic resources in maintaining self-esteem during treatment has been emphasized [15]. In Poland, issues related to life satisfaction and self-esteem in women with breast cancer have been explored; however, existing studies have predominantly employed cross-sectional designs or focused on patients after completion of surgical treatment. Previous Polish studies reported an average level of life satisfaction among women following breast cancer treatment, with age and place of residence identified as differentiating factors [16]. Physical activity was also associated with higher life satisfaction and better adaptation to the disease after oncological treatment [17]. The domain of self-esteem has most often been analyzed in the context of surgical treatment outcomes, with particular emphasis on its associations with sexual and psychosocial functioning, as well as body image and its acceptance [18,19].

Despite the growing body of research, there remains a lack of studies focusing directly on the course of chemotherapy, incorporating assessments conducted both before treatment initiation and after its completion, as well as considering potential sociodemographic factors such as financial status, marital status, and place of residence. The international literature indicates that psychological factors, including life satisfaction and self-esteem, may play a significant role in adaptation to cancer and represent potential targets for supportive interventions during systemic treatment [14,20]. Therefore, further investigation of these constructs in women undergoing chemotherapy for breast cancer appears warranted from both a scientific and clinical perspective.

The aim of this study was to assess changes in life satisfaction and self-esteem in women with breast cancer during neoadjuvant chemotherapy.

## 2. Materials and Methods

### 2.1. Study Area

The study was conducted in a specialized oncology hospital in Poland providing comprehensive cancer care, including systemic treatment and surgical management. The center serves both urban and rural populations, ensuring a diverse patient population representative of the regional setting. Participants were recruited consecutively from patients treated in the breast cancer unit of a specialized oncology hospital serving a regional referral population.

### 2.2. Study Design and Participants

A prospective observational study was conducted among 211 women diagnosed with breast cancer and treated at a specialized oncology hospital. All participants were informed about the purpose of the study and were assured of confidentiality, anonymity, and voluntary participation, with the right to withdraw at any stage of the study. Disease stage was considered within the population eligible for neoadjuvant treatment and was included as a clinical variable in the analyses.

Data were collected at two time points: one week before initiation of chemotherapy and three weeks after its completion. Participants completed the questionnaires independently in the presence of trained medical staff, ensuring completeness and reliability of the collected data. Questionnaires were administered in paper form at both study time points under supervision of trained medical personnel, who provided procedural support when needed. Additionally, participants completed a short questionnaire assessing sociodemographic characteristics.

The inclusion criteria were age ≥ 18 years, histologically confirmed breast cancer, and receipt of neoadjuvant chemotherapy consisting of at least four cycles based on taxanes and/or anthracyclines. Eligible patients were those qualified for neoadjuvant chemotherapy according to standard clinical indications, irrespective of biological subtype, provided they met treatment eligibility criteria at the study center.

The exclusion criteria included cognitive impairment preventing questionnaire completion, severe psychiatric disorders, concurrent malignancies, and incomplete data at either assessment time point.

### 2.3. Ethics

The study protocol was approved by the Bioethics Committee at the State University of Applied Sciences in Sanok (No. 3/2022) and by the Director of the Specialist Hospital. All procedures were conducted in accordance with the ethical standards of the institutional and national research committees and with the Declaration of Helsinki.

### 2.4. Tools

Two standardized questionnaires were used in the study: the Satisfaction with Life Scale (SWLS) and the Rosenberg Self-Esteem Scale (SES).

Life satisfaction, as a cognitive component of psychological well-being, was assessed using the *SWLS* developed by Diener et al. [10] and adapted into Polish by Juczyński [21]. The SWLS is a widely used instrument for measuring global life satisfaction as an indicator of subjective well-being and has been extensively applied in studies on QOL, mental health, and adaptation to chronic illness, including cancer populations.

The scale consists of five items rated on a 7-point Likert scale (1 = strongly disagree to 7 = strongly agree). The total score ranges from 5 to 35, with higher scores indicating greater life satisfaction.

Self-esteem was measured using the *SES*, developed by Rosenberg and adapted into Polish by Dzwonkowska et al. [22]. The SES assesses global self-esteem as a relatively stable evaluation of one’s self-worth, including both self-acceptance and perceived personal value.

The scale includes 10 items rated on a 4-point Likert scale (1 = strongly disagree to 4 = strongly agree), with both positively and negatively worded statements. Total scores range from 10 to 40, with higher scores indicating higher self-esteem. Negatively worded items were reverse-coded prior to analysis. Both instruments have been validated in the Polish population and are widely used in psychosocial and clinical research. No major feasibility or suitability concerns were identified in the present study sample.

### 2.5. Statistical Analysis

Statistical analyses were performed using STATISTICA software (version 13). The distribution of continuous variables was assessed using the Shapiro–Wilk test. As most variables deviated from normal distribution, non-parametric tests were applied.

Descriptive statistics were presented as means with 95% confidence intervals (95% CI), medians, standard deviations (SD), and ranges (minimum–maximum).

The significance of changes in psychometric measures after chemotherapy was assessed using the Wilcoxon signed-rank test. Results were considered statistically significant at *p* < 0.05 (in the text: *), with additional thresholds set at *p* < 0.01 (in the text: **) and *p* < 0.001 (in the text: ***).

Differences between groups according to variables (place of residence, marital status, financial situation and cancer stage) were evaluated using the Mann–Whitney U test. Associations between variables (life satisfaction—SWLS, self-esteem—SES, age, body mass index, and number of children) were analyzed using Spearman’s rank correlation coefficient. All statistical tests were two-tailed. Selected sociodemographic variables potentially related to psychosocial outcomes, including marital status and financial status, were considered in subgroup analyses.

### 2.6. The Use of GenAI

Generative artificial intelligence tools were used solely for language editing and improvement of clarity, grammar, and style. No AI tools were used to generate scientific content, analyze data, or interpret results.

## 3. Results

Detailed sociodemographic characteristics of the study population are presented in Table 1.

For the SWLS, the mean level of life satisfaction before chemotherapy was 21.6 points, indicating slight life satisfaction. After chemotherapy, the mean SWLS score decreased to 18.7 points, corresponding to slight dissatisfaction. The mean change was −2.9 points, indicating a statistically significant decline in life satisfaction following treatment (*p* < 0.001).

For the SES, the mean self-esteem score before chemotherapy was 29.4 points, corresponding to an average level of self-esteem. After chemotherapy, the mean score decreased to 27.8 points, remaining within the average range but closer to its lower bound. The observed mean change was −1.6 points and was statistically significant (*p* < 0.001), indicating a reduction in self-esteem after treatment (Table 2).

The direction of changes in life satisfaction (SWLS) and self-esteem (SES) after chemotherapy compared with pre-treatment values is presented in Table 3.

In terms of life satisfaction (SWLS), a decrease was observed in the majority of participants (60.7%, *N* = 128). No change was reported in 24.2% of patients (*N* = 51), while an increase in life satisfaction was observed in 15.2% of the study group (*N* = 32).

**Figure 1:**
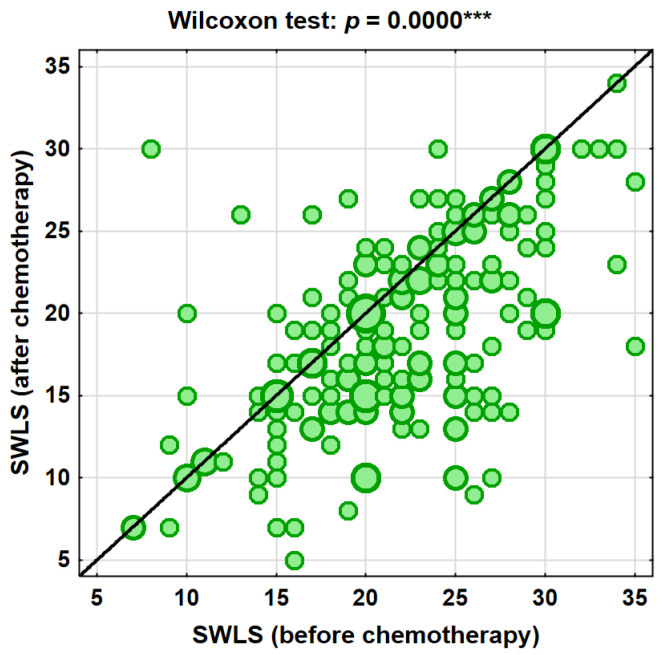
Changes in life satisfaction (SWLS) after chemotherapy (*** *p* < 0.001).

**Figure 2:**
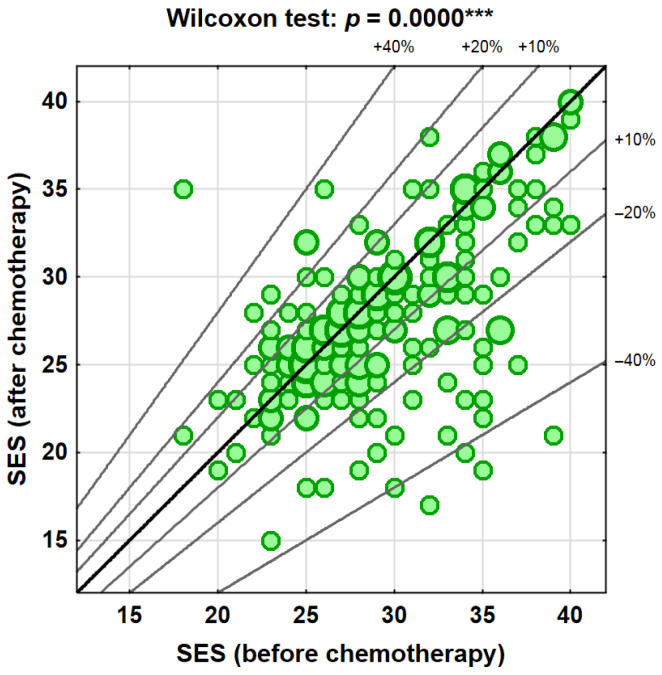
Changes in self-esteem (SES) after chemotherapy (*** *p* < 0.001).

Analysis of self-esteem (SES) showed that 50.2% of participants (*N* = 106) experienced a decrease following chemotherapy. No change was observed in 23.7% of patients (*N* = 50), whereas an increase in self-esteem was reported in 26.1% of participants (*N* = 55) (Table 3, Figure 1 and Figure 2). Importantly, a decrease in life satisfaction was observed in more than half of the participants (60.7%), and a reduction in self-esteem in 50.2% of patients, indicating that the observed changes affected a substantial proportion of the study population.

SWLS and SES measures were significantly correlated in all analyzed time points, and a significant correlation was also observed for changes during chemotherapy. The correlation was slightly stronger before chemotherapy (rS = 0.49) than after chemotherapy (rS = 0.39). Higher life satisfaction was associated with higher self-esteem, and a decrease in life satisfaction following chemotherapy was also associated with a decrease in self-esteem (Table 4).

The relationship between sociodemographic factors and the level of life satisfaction assessed using the SWLS is presented in Table 5. Place of residence did not significantly differentiate life satisfaction (SWLS) among women undergoing chemotherapy, nor the magnitude of decline in life satisfaction during treatment (*p*-values for all comparisons were well above 0.05). Similar findings were observed for marital status. Some differences were noted with regard to financial situation. Women with a poorer financial status reported lower life satisfaction both before (19.3 points) and after chemotherapy (16.2 points). The difference observed before treatment approached statistical significance (*p* = 0.0709), while after chemotherapy it reached statistical significance (*p* = 0.0305). A decline in life satisfaction following chemotherapy was observed in both groups; however, the magnitude of this decrease did not differ significantly between them (*p* = 0.1977).

Additional analyses indicated that financial status was associated with differences in post-treatment life satisfaction and self-esteem, suggesting potentially greater psychosocial vulnerability among patients with poorer economic conditions. In contrast, marital status and place of residence were not associated with significantly different declines in these outcomes.

Women living in urban areas experienced a slightly greater decrease in self-esteem after chemotherapy compared to those living in rural areas (2.0 vs. 1.3 points). This difference did not reach statistical significance (*p* = 0.0637), although a trend was observed. Participants who were in a relationship had higher self-esteem before chemotherapy; however, this difference did not reach statistical significance (29.7 vs. 28.0; *p* = 0.0773). Notably, women in a relationship also demonstrated a greater decline in self-esteem following chemotherapy compared to single women (a decrease of 1.9 vs. 0.6 points), with the difference not reaching statistical significance (*p* = 0.0520), although a trend was observed. Women with a poor financial situation had lower self-esteem before chemotherapy and experienced a greater decline following treatment. Although these differences were not statistically significant, their cumulative effect resulted in significantly lower post-chemotherapy self-esteem among women with poor financial status compared to those with a good financial situation (25.2 vs. 28.1 points; *p* = 0.0016 **) (Table 6).

To assess the relationships between sociodemographic variables and psychological functioning, Spearman’s rank correlation analysis was performed. Associations between age, body mass index (BMI), and number of children and life satisfaction (SWLS) as well as self-esteem measured using the SES were analyzed at three time points: before chemotherapy, after its completion, and as changes in these measures. In terms of life satisfaction, no statistically significant correlations were observed with the analyzed sociodemographic variables before the initiation of treatment (all *p* > 0.05). After chemotherapy, a statistically significant but weak negative correlation was found between BMI and life satisfaction (ρ = −0.16; *p* = 0.0187), indicating that higher BMI was associated with lower life satisfaction. No significant associations were observed between life satisfaction and age or number of children. Similarly, no significant correlations were found between changes in life satisfaction and any of the analyzed variables.

With regard to self-esteem, no statistically significant associations with age, BMI, or number of children were observed before chemotherapy (*p* > 0.05). However, after treatment, significant negative correlations were identified between self-esteem and age (ρ = −0.25; *p* = 0.0002) as well as between self-esteem and BMI (ρ = −0.20; *p* = 0.0033). These findings indicate that older age and higher BMI were associated with lower self-esteem after chemotherapy. The number of children was not significantly associated with self-esteem. Analysis of changes in self-esteem during treatment did not reach statistical significance; however, trends approaching significance were observed for age and BMI (*p* = 0.054 and *p* = 0.066, respectively), suggesting a potential influence of these variables on the dynamics of self-esteem changes (Table 7).

No statistically significant differences in life satisfaction or self-esteem were observed between patients with stage I and stage II–III disease at either time point, nor in the magnitude of change after chemotherapy (Table 8).

## 4. Discussion

Neoadjuvant chemotherapy (NAC) is currently widely used in the treatment of breast cancer. In early-stage disease, it enables high response rates, which translate into an increased likelihood of breast-conserving surgery and improved long-term prognosis in patients achieving pathological complete response [23].

However, NAC is also associated with numerous adverse effects affecting multiple organ systems. Following chemotherapy, patients typically require surgical treatment. Treatment-related side effects and deterioration in psychosocial well-being may have broader clinical relevance during treatment; however, possible associations with surgical tolerance or delays in surgical intervention were not evaluated in the present study. Both the initial phase of therapy and the months following its completion represent a period of substantial physical and psychological burden, contributing to adaptation difficulties and reduced QOL [24]. Therefore, anticipated levels of life satisfaction and self-esteem should be considered an important component of the decision-making process for both patients and clinicians when planning NAC. However, it should be noted that the present findings are based on an assessment conducted only three weeks after completion of neoadjuvant chemotherapy. Therefore, the observed changes in life satisfaction and self-esteem are likely to reflect short-term treatment-related effects, including acute toxicity and preoperative psychological distress, rather than stable long-term psychosocial outcomes.

The present analysis was intentionally based on a pre–post comparison design, which allows for a clear assessment of overall changes during the neoadjuvant chemotherapy period but does not account for the complexity of clinical and treatment-related factors that may influence psychosocial outcomes.

In particular, clinical heterogeneity related to tumor subtype, disease stage, and treatment strategy may have influenced the observed changes in psychosocial outcomes. Patients with more aggressive tumor biology or those requiring more intensive multimodal treatment may experience a greater psychological burden during therapy. As these variables were not included in the present analysis, the results should be interpreted as reflecting overall trends rather than subgroup-specific effects.

The present study demonstrated a statistically significant decrease in both life satisfaction (SWLS) and self-esteem (SES) following neoadjuvant chemotherapy, indicating a measurable impact of systemic treatment on psychosocial functioning. Mean life satisfaction declined from slight satisfaction before treatment to slight dissatisfaction after chemotherapy, while self-esteem decreased significantly but remained within the average range. These findings suggest a noticeable psychological burden associated with treatment. To our knowledge, this study is among the few that assess both life satisfaction and self-esteem in women with breast cancer and compare these measures before and after NAC. Importantly, the timing of the post-treatment assessment should be considered when interpreting these findings. Psychological outcomes were evaluated only three weeks after completion of chemotherapy, a period during which acute treatment-related toxicity may still be present and surgical treatment has not yet been performed. Therefore, the observed changes are likely to reflect short-term treatment burden and the immediate post-chemotherapy/preoperative phase rather than long-term psychosocial adaptation.

Although the observed changes in life satisfaction and self-esteem were statistically significant, their clinical relevance should be interpreted with caution. The decrease in life satisfaction was accompanied by a shift in mean SWLS scores from a level corresponding to slight satisfaction before treatment toward a less favorable level after chemotherapy. In contrast, although self-esteem decreased significantly, the mean SES score remained within the average range, suggesting a more modest clinical impact.

Furthermore, it should be noted that established minimal clinically important differences for the SWLS and SES in women undergoing neoadjuvant chemotherapy are not well defined. Therefore, the clinical significance of the observed changes should be interpreted in the context of both statistical results and overall score distribution.

Our findings also suggest that socioeconomic factors, particularly financial status, may influence vulnerability to deterioration in psychosocial outcomes during neoadjuvant chemotherapy. This observation may help identify patients who could particularly benefit from supportive interventions.

The available literature provides relatively limited data on psychosocial functioning in women with breast cancer assessed both before and after NAC. Most studies focus primarily on QOL, while aspects such as life satisfaction and self-esteem remain underexplored, despite being key indicators of psychological well-being. Importantly, health-related life satisfaction is closely linked to overall QOL; therefore, it is reasonable to interpret the present findings in the context of studies evaluating QOL in patients undergoing chemotherapy.

A systematic review by Zhao et al. demonstrated that patients undergoing chemotherapy experience a significant decline in QOL, particularly in emotional, physical, and global functioning domains [25]. Similarly, Pelzer et al., in a prospective multicenter study, reported increasing fatigue and deterioration in QOL during NAC, highlighting its multidimensional impact on patient functioning [4].

Carvalho et al. showed that NAC is associated with worsening QOL, increased depressive symptoms, and disturbances in body image. The decline in global life evaluation may result from the accumulation of somatic symptoms such as fatigue, nausea, and cognitive impairment, which directly affect subjective well-being [26]. Additionally, Santos et al., comparing adjuvant and neoadjuvant chemotherapy, reported moderate levels of QOL and psychosocial functioning in both groups, indicating that intensive systemic treatment constitutes a substantial psychological burden regardless of timing [27]. Rodríguez-González et al. further confirmed that systemic therapy is associated with deterioration in QOL and psychological well-being in both early and advanced disease [28].

Several mechanisms may explain the observed changes in life satisfaction and self-esteem during neoadjuvant chemotherapy. First, treatment burden and cumulative physical side effects, such as fatigue, nausea, and cognitive impairment, may directly affect patients’ subjective well-being. Second, financial toxicity related to cancer treatment, including direct and indirect costs, may contribute to increased stress and reduced life satisfaction. Third, individual coping resources and psychological resilience may play a key role in moderating the impact of treatment-related stressors. Patients with more adaptive coping strategies may better maintain psychological well-being despite the challenges associated with intensive systemic therapy.

Another key finding of the present study was the significant decrease in self-esteem following chemotherapy. This result is consistent with previous research indicating that self-esteem is particularly sensitive to the effects of oncological treatment. Tsai et al. demonstrated that lower self-esteem is a significant predictor of poorer psychological adjustment in breast cancer patients and is associated with increased psychological distress and reduced QOL [14].

A potential mechanism underlying the decline in self-esteem involves changes in body image and sexual functioning, which frequently accompany systemic treatment. Prates et al. emphasized that alterations in physical appearance, hair loss, and functional limitations significantly affect self-perception in women treated for breast cancer [5]. Moreover, Neves et al. reported that NAC may negatively impact sexual QOL, an important yet often underestimated component of psychological well-being and self-esteem [29]. Cobo-Cuenca et al. further demonstrated that self-esteem is a key determinant of life satisfaction in women with breast cancer, which is directly supported by the findings of the present study [30].

The significant positive correlation between life satisfaction and self-esteem observed both before and after chemotherapy indicates a strong interrelationship between these constructs and their shared foundation within psychological well-being. This finding is consistent with the results of Cieślak et al., who showed that psychological adaptation to illness and intrapsychic resources are strongly associated with life satisfaction in women with breast cancer [31]. Soria-Reyes et al. further demonstrated that psychological resources such as hope and vitality are important predictors of life satisfaction. From a practical perspective, this suggests that women who maintain optimism, energy, and goal-oriented behavior are more likely to achieve higher levels of life satisfaction [32].

Our findings also indicate that financial situation is an important factor differentiating both life satisfaction and self-esteem after chemotherapy, which is consistent with previous studies highlighting the role of sociodemographic factors in shaping patient well-being [16]. At the same time, the literature emphasizes the crucial role of social support. Aprilianto et al. demonstrated that family support significantly enhances self-esteem in patients undergoing chemotherapy, acting as a protective factor in the adaptation process [33].

The lack of significant differences related to place of residence and marital status observed in the present study may suggest that during intensive treatment, clinical and psychological factors play a more dominant role than classical sociodemographic variables. It is likely that these factors play an important role in shaping individual psychosocial responses to treatment and should be addressed in future studies. The analyses performed in this study were limited to selected sociodemographic variables and should therefore be interpreted as exploratory rather than as identifying the main determinants of psychosocial outcomes.

The obtained results highlight the need for routine assessment of psychological well-being during oncological treatment. Indicators such as life satisfaction and self-esteem may serve as important predictors of adaptation to illness and overall QOL. An integrated therapeutic approach, including psycho-oncological support, may help mitigate the negative effects of treatment and improve overall patient well-being.

The relatively short and fixed follow-up period applied in this study (three weeks after chemotherapy completion) should be considered when interpreting the results. The findings reflect short-term changes during the treatment phase and may not capture longer-term psychosocial adaptation. Future studies with extended and variable follow-up periods are needed to better understand the trajectory of psychological outcomes over time.

From a clinical perspective, routine psychological screening before and during neoadjuvant treatment may help identify patients at risk of deteriorating psychosocial well-being. Patients showing substantial declines in SWLS or SES may benefit from referral for psycho-oncological support. Integrating such assessment into multidisciplinary oncology care may improve supportive management.

The findings have important clinical implications, suggesting that psychosocial changes during neoadjuvant chemotherapy should be considered as part of comprehensive cancer care. Routine monitoring of well-being may help identify vulnerable patients early and support timely supportive interventions during treatment.

### Study Limitations

The present study has several limitations. First, it was conducted in a single center, which may limit the generalizability of the findings. Second, the lack of long-term follow-up prevents assessment of the persistence of observed changes in life satisfaction and self-esteem over time. Additionally, potential confounding factors, such as psychological support or comorbidities, were not fully controlled.

Furthermore, the short interval between chemotherapy completion and the second assessment (three weeks) limits the ability to evaluate long-term psychosocial outcomes, and the results should therefore be interpreted as reflecting short-term changes during the treatment period.

Importantly, the analysis did not include several clinically relevant variables that may influence psychosocial outcomes during neoadjuvant chemotherapy, such as tumor subtype, treatment response, chemotherapy regimen, treatment-related toxicity, or planned surgical management. Moreover, the lack of detailed clinical stratification limits the ability to assess whether the observed changes in life satisfaction and self-esteem differed across clinically relevant patient subgroups. These factors may act as important effect modifiers and could contribute to inter-individual variability in psychosocial outcomes. Therefore, the observed changes should be interpreted as overall trends during treatment rather than as effects attributable to specific clinical factors.

Additionally, data on psychological support, social support systems, and psychiatric comorbidities were not collected, which may have influenced the observed psychosocial outcomes.

These factors may significantly influence psychosocial outcomes and could partly explain the variability observed in life satisfaction and self-esteem.

However, additional analyses performed for disease stage did not demonstrate significant differences in either life satisfaction or self-esteem, nor in their changes over time, suggesting that the observed decline may be relatively consistent across different levels of disease advancement. Nevertheless, the potential influence of other unmeasured clinical variables cannot be excluded and should be addressed in future research.

Potential confounding factors such as social support, comorbidities, and prior psychological resources were not comprehensively controlled in this study and should be considered in interpreting the findings.

## 5. Conclusions

In this prospective study, life satisfaction and self-esteem decreased significantly over the course of the neoadjuvant chemotherapy period. These changes appear to be moderate in magnitude, with self-esteem remaining within the average range after treatment, suggesting a limited clinical impact. The significant positive correlation between life satisfaction and self-esteem, observed both before and after chemotherapy, suggests that these constructs are closely interrelated and may serve as complementary indicators of psychological well-being in this patient population.

Financial status emerged as an important factor differentiating levels of life satisfaction and self-esteem after treatment, highlighting the role of socioeconomic conditions in psychosocial adaptation to cancer. Furthermore, older age and higher body mass index were associated with lower self-esteem following chemotherapy, suggesting that certain patient groups may be more vulnerable to deterioration in psychological functioning during treatment.

These findings underline the need for routine monitoring of life satisfaction and self-esteem during systemic treatment of breast cancer as important components of comprehensive patient assessment. The integration of psycho-oncological support and targeted interventions aimed at improving psychological well-being should be considered a standard element of care for women undergoing neoadjuvant chemotherapy, as it may help mitigate negative psychosocial effects and support adaptation to the disease. Given the short follow-up period, these findings should be interpreted as short-term effects rather than evidence of sustained long-term changes.

### Future Directions

Future research should focus on longitudinal assessment of psychological outcomes beyond the immediate post-chemotherapy period, including post-surgical and survivorship phases. Additionally, future studies should incorporate detailed clinical variables, such as tumor subtype, disease stage, treatment response, and type of therapy, to allow for subgroup analyses and better understanding of factors influencing psychosocial outcomes. There is also a need to evaluate the effectiveness of targeted psycho-oncological interventions aimed at maintaining life satisfaction and self-esteem during cancer treatment.

## Figures and Tables

**Table 1:** Participant sociodemographic characteristics.

Parameter		Years
Age	Mean	56.2
	SD	11.0
	Median	56
	Min.	32
	Max.	84
Parameter		*N* (%)
Residence	Rural	117 (55.5%)
	Urban	94 (44.5%)
Marital status	In relationship	175 (82.9%)
	Single	36 (17.1%)
Education	Primary/Secondary	11 (5.2%)
	Vocational	44 (20.9%)
	Secondary/Post-secondary	91 (43.1%)
	Higher	65 (30.8%)
Employment status	Working	104 (49.3%)
	Retired/disabled	39 (18.5%)
	Unemployed	64 (30.3%)
	Other	4 (1.9%)
Financial situation	Very good	11 (5.2%);
	Good	174 (82.5%);
	Bad	26 (12.3%)

**Table 2:** Changes in Life Satisfaction (SWLS) and Self-Esteem (SES) Before and After Neoadjuvant Chemotherapy.

Scale	Study Timeline	Mean (with 95% CI)	Median	SD	Min	Max
SWLS	before chemotherapy	21.6 (20.9; 22.4)	22	5.7	7	35
	after chemotherapy	18.7 (17.9; 19.5)	19	5.8	5	34
	change (*p* = 0.0000 ***)	−2.9 (−3.6; −2.2)	−2	5.2	−17	22
	before chemotherapy	29.4 (28.8; 30.1)	29	4.8	18	40
SES	after chemotherapy	27.8 (27.1; 28.4)	27	4.8	15	40
	change (*p* = 0.0000 ***)	−1.6 (−2.2; −1.0)	−1	4.4	−18	17

*p*-value calculated using the Wilcoxon signed-rank test; *** *p* < 0.001.

**Table 3:** Changes in Life Satisfaction (SWLS) and Self-Esteem (SES) after Chemotherapy.

Scale	Direction of Change	*N*	%
SWLS	decrease	128	60.7%
	no change	51	24.2%
	increase	32	15.2%
SES	decrease	106	50.2%
	no change	50	23.7%
	increase	55	26.1%

**Table 4:** Correlation between Self-Esteem and Life Satisfaction—Spearman’s Rank Correlation Coefficient (rS) with Statistical Significance (*p*).

Life Satisfaction	Rosenberg Self-Esteem Scale (SES)		
	Before Chemotherapy	After Chemotherapy	Change
SWLS	*r*_S_ 0.49 (*p* = 0.0000 ***)	*r*_S_ 0.39 (*p* = 0.0000 ***)	*r*_S_ 0.34 (*p* = 0.0000 ***)

*** *p* < 0.001.

**Table 5:** Life Satisfaction (SWLS) and Sociodemographic Characteristics of the Study Population.

Variable		Sociodemographic Characteristic				
		Mean (95% CI)	Median	Mean (95% CI)	Median	*p*
		*Residence*				
		Rural (*N* = 117)		Urban (*N* = 94)		
SWLS	before chemotherapy	21.6 (20.6; 22.7)	22	21.7 (20.5; 22.9)	20.5	0.7501
	after chemotherapy	18.4 (17.4; 19.5)	17	19.0 (17.8; 20.2)	19.5	0.4504
	change	−3.2 (−3.9; −2.4)	−2	−2.7 (−3.9; −1.4)	−2	0.5628
		***Marital Status***				
		**Single (*N* = 36)**		**In Relationship (*N* = 175)**		
SWLS	before chemotherapy	20.4 (18.3; 22.4)	20	21.9 (21.1; 22.7)	22	0.1494
	after chemotherapy	18.0 (16.2; 19.8)	17.5	18.8 (18.0; 19.7)	19	0.5410
	change	−2.4 (−3.8; −1.0)	−1	−3.1 (−3.9; −2.3)	−2	0.3049
		***Financial Situation*** **^a^**				
		**Good (*N* = 174)**		**Bad (*N* = 26)**		
SWLS	before chemotherapy	22.0 (21.2; 22.8)	22	19.3 (16.5; 22.2)	20.5	0.0769
	after chemotherapy	19.1 (18.2; 19.9)	19	16.2 (13.6; 18.7)	16	0.0305 *
	change	−2.9 (−3.6; −2.2)	−2	−3.2 (−6.5; 0.1)	−3.5	0.1977

^a^ Due to the small number of participants with a very good financial situation, this group was not included in the statistical analysis; * *p* < 0.05.

**Table 6:** Self-Esteem (SES) by Sociodemographic Characteristics.

Variable		Sociodemographic Characteristic				
		Mean (95% CI)	Median	Mean (95% CI)	Median	*p*
		*Residence*				
		Rural (*N* = 117)		Urban (*N* = 94)		
SES	before chemotherapy	29.0 (28.2; 29.8)	29	29.9 (28.9; 31.0)	29	0.2470
	after chemotherapy	27.7 (26.8; 28.5)	27	27.9 (26.9; 29.0)	27	0.9474
	change	−1.3 (−2.1; −0.6)	0	−2.0 (−3.0; −1.1)	−1	0.0637
		***Marital Status***				
		**Single (*N* = 36)**		**In Relationship (*N* = 175)**		
SES	before chemotherapy	28.0 (26.6; 29.5)	27	29.7 (29.0; 30.4)	29	0.0773
	after chemotherapy	27.4 (26.1; 28.7)	27	27.9 (27.1; 28.6)	27	0.7139
	change	−0.6 (−1.8; 0.7)	0	−1.9 (−2.5; −1.2)	−1	0.0520
		***Financial Situation*** **^a^**				
		**Good (*N* = 174)**		**Bad (*N* = 26)**		
SES	before chemotherapy	29.6 (28.9; 30.3)	29	28.4 (26.3; 30.5)	27	0.1759
	after chemotherapy	28.1 (27.5; 28.8)	28	25.2 (23.5; 27.0)	25	0.0016 **
	change	−1.4 (−2.0; −0.8)	−1	−3.2 (−5.7; −0.7)	−0.5	0.4745

^a^ Due to the small number of participants with a very good financial situation, they were not included in the statistical analysis; ** *p* < 0.01.

**Table 7:** Life Satisfaction (SWLS) and Self-Esteem (SES) in relation to age, BMI, and number of children.

Variable			
Life Satisfaction (SWLS)	Age (Years)	BMI	Number of Children
	*r*_S_, (*p*)	*r*_S_ (*p*)	*r*_S_, (*p*)
before chemotherapy	−0.10, (*p* = 0.1352)	−0.08, (*p* = 0.2398)	−0.08, (*p* = 0.2305)
after chemotherapy	−0.01, (*p* = 0.8428)	−0.16, (*p* = 0.0187 *)	−0.08, (*p* = 0.2221)
change	0.07, (*p* = 0.3202)	−0.07 (*p* = 0.3072)	−0.03, (*p* = 0.6934)
**Rosenberg Self-Esteem Scale (SES)**			
before chemotherapy	−0.11, (*p* = 0.1251)	−0.06, (*p* = 0.4250)	0.00, (*p* = 0.9879)
after chemotherapy	−0.25, (*p* = 0.0002 ***)	−0.20, (*p* = 0.0033 **)	−0.08, (*p* = 0.2551)
Change	−0.13, (*p* = 0.0543)	−0.13, (*p* = 0.0661)	−0.08, (*p* = 0.2686)

* *p* < 0.05, ** *p* < 0.01, ***p < 0.001.

**Table 8:** Comparison of life satisfaction (SWLS) and self-esteem (SES) before and after neoadjuvant chemotherapy according to disease stage.

Scale	Assessment Time Point	Cancer Stage				*p*
		I (*N* = 31)		II–III (*N* = 180)		
		Mean (95% CI)	Median	Mean (95% CI)	Median	
SES	before chemotherapy	29.2 (28.5; 29.9)	28	30.5 (28.7; 32.3)	30	0.1254
	after chemotherapy	27.8 (27.1; 28.4)	27	27.9 (25.9; 30.0)	28	0.5224
	change	−1.5 (−2.1; −0.9)	0	−2.5 (−4.7; −0.4)	−2	0.1958
SWLS	before chemotherapy	21.7 (20.8; 22.5)	22	21.4 (19.4; 23.4)	21	0.9095
	after chemotherapy	18.7 (17.8; 19.6)	19	18.8 (16.8; 20.8)	19	0.9926
	change	−3.0 (−3.7; −2.3)	−2	−2.6 (−4.8; −0.4)	−2	0.8254

## Data Availability

Data can be obtained by contacting the corresponding author.
